# Biomechanical Measurement Error Can Be Caused by Fujifilm Thickness: A Theoretical, Experimental, and Computational Analysis

**DOI:** 10.1155/2017/4310314

**Published:** 2017-08-16

**Authors:** Ahmed Sarwar, Simli Srivastava, Chris Chu, Alan Machin, Emil H. Schemitsch, Habiba Bougherara, Zahra S. Bagheri, Radovan Zdero

**Affiliations:** ^1^Department of Mechanical and Industrial Engineering, Ryerson University, Toronto, ON, Canada M5B 2K3; ^2^Orthopaedic Biomechanics Lab, Victoria Hospital, London, ON, Canada N6A 5A5; ^3^Department of Surgery, Western University, London, ON, Canada N6A 5A5; ^4^iDAPT Centre for Rehabilitation Research, Toronto Rehab Institute, Toronto, ON, Canada M5G 2A2; ^5^Department of Mechanical and Materials Engineering, Western University, London, ON, Canada N6A 5A5

## Abstract

This is the first study to quantify the measurement error due to the physical thickness of Fujifilm for several material combinations relevant to orthopaedics. Theoretical and experimental analyses were conducted for cylinder-on-flat indentation over a series of forces (750 and 3000 N), cylinder diameters (0 to 80 mm), and material combinations (metal-on-metal, MOM; metal-on-polymer, MOP; metal-on-bone, MOB). For the scenario without Fujifilm, classic Hertzian theory predicted the true line-type contact width as *W*_*O*_ = {(8FD_cyl_)/(*πL*_cyl_)[(1 − *ν*_cyl_^2^)/*E*_cyl_ + (1 − *ν*_flat_^2^)/*E*_flat_]}^1/2^, where *F* is compressive force, *D*_cyl_ is cylinder diameter, *L*_cyl_ is cylinder length, *ν*_cyl_ and *ν*_flat_ are cylinder and flat Poisson's ratios, and *E*_cyl_ and *E*_flat_ are cylinder and flat elastic moduli. For the scenario with Fujifilm, experimental measurements resulted in contact widths of *W*_*F*_ = 0.1778 × *F*^0.2273^ × *D*^0.2936^ for MOM tests, *W*_*F*_ = 0.0449 × *F*^0.4664^ × *D*^0.4201^ for MOP tests, and *W*_*F*_ = 0.1647 × *F*^0.2397^ × *D*^0.3394^ for MOB tests, where *F* is compressive force and *D* is cylinder diameter. Fujifilm thickness error ratio *W*_*F*_/*W*_*O*_ showed a nonlinear decrease versus cylinder diameter, whilst error graphs shifted down as force increased. Computational finite element analysis for several test cases agreed with theoretical and experimental data, respectively, to within 3.3% and 1.4%. Despite its wide use, Fujifilm's measurement errors must be kept in mind when employed in orthopaedic biomechanics research.

## 1. Introduction

Various experimental methods exist in orthopaedic biomechanics research for measuring interfacial contact areas of human and artificial joints [1]. These techniques can be characterized as direct contact substances (e.g., castings, dyes), electrical resistivity sensors (e.g., piezoelectric transducers, resistive ink sensors, and radiotelemetry), mechanically deformable films (e.g., microindentation pads, chemically sensitive pads), and nonintrusive techniques (e.g., radiography, ultrasound) [1]. A very popular approach is pressure sensitive Fujifilm [1–9] in which a reaction occurs between an acid indicator and an acid that are suspended in an absorbent and flexible sheet. The two pads are stacked to form a 0.2 mm thick composite placed between two articulating surfaces. When pressure is applied to the pad, the reactants combine to produce a color change proportional to pressure. The perimeter of the contact patch encompasses the maximum contact area engaged. Fujifilm is simple to use, inexpensive, available, and nontoxic and provides quick results. However, it is limited to two-dimensional quasi-static in vitro use, there is a minimum pressure threshold to detect contact, spatial resolution is restricted, it is sensitive to shear, and there is difficulty in accurately detecting pressure gradients near the edges of the contact area. Despite this and the availability of K-scan real-time thin film technology [10–13], Fujifilm continues to enjoy wide use in orthopaedic biomechanics research. Some studies have quantified Fujifilm's limitations due to image processing methods, pressure threshold requirements, and gradients along the edge of the contact area [13–19], but no prior investigators determined or predicted measurement error due to the Fujifilm's finite physical thickness during contact area measurement tests for a number of material combinations relevant to orthopaedics. This study determined the error due to the physical thickness of Fujifilm at the interface of two-body articulations of metal-on-metal (MOM), metal-on-polymer (MOP), and metal-on-bone (MOB) using a cylinder-on-flat geometry as “proof of principle.”

## 2. Methods

### 2.1. General Approach

**Figure 1:**
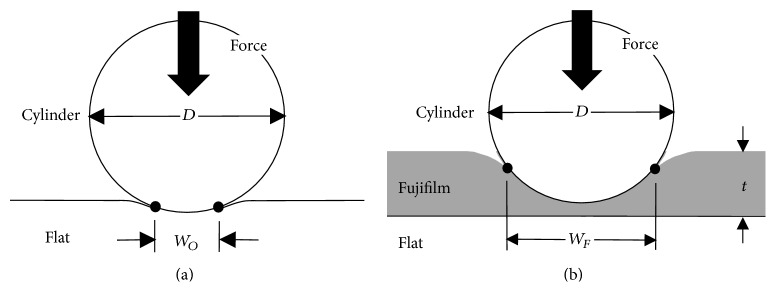
Cylinder-on-flat contact widths. (a) Contact without Fujifilm and (b) contact with Fujifilm. The third dimension (into the page) of cylinder and flat substrate lengths are not shown. Diagrams are not to scale. *W*_*O*_ is contact width without Fujifilm, *W*_*F*_ is contact width with Fujifilm, *D* is cylinder diameter, and *t* is Fujifilm thickness.

Using the common orthopaedic material combinations of MOM, MOP, and MOB, the measurement error due to the physical thickness of Fujifilm was quantified using a cylinder-on-flat configuration as a “proof of principle” (Figure 1). This simulated in a rudimentary way the noncongruent interfacial contact and material combinations in various total knee replacements (e.g., metal-on-polymer Genesis, Miller/Galante, Orthomet Plus, PCA Modular, PFC, and Whiteside Ortholoc II implants) [6, 20], total hip replacements (e.g., metal-on-metal hip resurfacings or metal-on-polymer traditional hip implants), and fracture fixation devices (metal cable-on-bone, metal nail-on-bone, metal plate-on-bone, etc.). Presently, classic Hertzian contact theory predicted the true interfacial line-type contact width *W*_*O*_ created for cylinder-on-flat indentation in the absence of Fujifilm, corresponding experiments measured interfacial line-type contact width *W*_*F*_ using Fujifilm, and computational finite element analysis was used to confirm theoretical and experimental accuracy for several test cases without and with Fujifilm. Contact error was then calculated by comparing results without and with Fujifilm.

### 2.2. Theoretical Analysis: Contact without Fujifilm

All experimental methods for measuring interfacial contact area have resolution limits and/or physically disturb the interface and, thus, could not be used to quantify true cylinder-on-flat contact width for the scenario without Fujifilm [1]. Consequently, classic Hertzian contact mechanics theory was used for the case without Fujifilm (Figure 1(a)). Hertzian formulas are valid for quasi-static normal loads, nonconforming surfaces, smooth surfaces with frictionless contact, and small linear elastic deformations in which the contact width is much smaller than the cylinder diameter [3, 21, 22]. Hertzian analysis was applied under the following conditions. First, cylinder diameters of 0 to 80 mm covered a wide range of orthopaedic applications (the mid-shaft diameter is about 30 mm for an adult femur, the head diameter is about 42 mm for a hip resurfacing implant, the diameter of curvature is about 50 mm for the femoral metal component of a total knee replacement, etc.). Second, quasi-static compressive forces of 750 and 3000 N, respectively, simulated 1x and 4x body weight for a 75 kg person, which occur at lower extremity joints during walking [23]. Third, the material properties of elastic modulus and Poisson's ratio were based on steel, ultrahigh molecular weight polyethylene (UHMWPE), and artificial cortical bone, respectively, for MOM, MOP, and MOB configurations, to match later experiments. Finally, the Hertzian equation for cylinder-on-flat indentation was used in the following form:

(1) $$\begin{array}{c} W_{O}=\sqrt{\left(\frac{8FD_{cyl}}{\pi L_{cyl}}\right)\left(\frac{1-\nu_{cyl}^{2}}{E_{cyl}}+\frac{1-\nu_{flat}^{2}}{E_{flat}}\right)}, \end{array}$$

where *W*_*O*_ is contact width without Fujifilm, *F* is compressive force (i.e., 750 or 3000 N), *D*_cyl_ is cylinder diameter (i.e., 0 to 80 mm), *L*_cyl_ is cylinder length that mimics later experiments (i.e., 75 mm of cylinder length was in contact for MOM tests, but 50 mm of cylinder length was in contact for MOP and MOB tests), *ν*_cyl_ is Poisson's ratio for a steel cylinder (0.31), *ν*_flat_ is Poisson's ratio for steel (0.31), UHMWPE (0.4), or artificial cortical bone (0.3) flat substrate, *E*_cyl_ is elastic modulus for a steel cylinder (210 GPa), and *E*_flat_ is elastic modulus for a steel (210 GPa), UHMWPE (0.9 GPa), or artificial cortical bone (16.7 GPa) flat substrate [3, 21, 22, 24, 25].

### 2.3. Experimental Analysis: Contact with Fujifilm

#### 2.3.1. Fujifilm Preparation

“Ultralow” Fujifilm had a 0.2 mm total thickness, a 0.19 MPa (i.e., 28 psi) minimum pressure sensing threshold, and an operating temperature range of 20–35°C; it was cut into 25 mm × 25 mm squares (Pressuremetrics, Whitehouse Station, NJ, USA). The Fujifilm acid layer was always placed on top of the indicator layer for all indentation tests. Each Fujifilm square was used only once for each indentation test.

#### 2.3.2. Cylinder Preparation

Metal cylinders were manufactured from steel with diameters of 1.6 mm (1/16 inch), 12.7 mm (1/2 inch), 25.4 mm (1 inch), 50.8 mm (2 inch), and 76.2 mm (3 inch). Vernier digital calipers (Model #58-6800-4, Mastercraft, Toronto, ON, Canada) were used to measure an average diameter tolerance of +/− 25 *μ*m, whilst a surface profilometer (Surfcom 112B, Browne & Sharpe, North Kingstown, RI, USA) was used to measure an average surface roughness of 0.857 *μ*m (i.e., 0.005% of the diameter). Metal cylinders were 75 mm long, that is, longer than the Fujifilm square width, so that even pressure distribution across the film was generated and each cylinder engaged the same length of film. Each of the five metal cylinders was used multiple times during the study.

#### 2.3.3. Flat Substrate Preparation

Flat substrates to be indented were made from metal (i.e., steel), polymer (i.e., UHMWPE), and bone (i.e., artificial cortical bone) to match earlier theoretical analysis. The metal plate was 5 mm thick × 150 mm wide × 150 mm long having an average surface roughness of 1.907 *μ*m (i.e., 0.039% of plate thickness); it was used for all MOM tests. A series of 30 polymer plates (Model # Jaytrex 1000, Johnston Industrial Plastics, Toronto, ON, Canada) were 25 mm thick × 50 mm wide × 50 mm long having an average surface roughness of 0.859 *μ*m (i.e., 0.003% of plate thickness); they were each used only once for MOP tests. A series of 30 bone plates made from glass-filled epoxy resin (Model # 1523-22, Sawbones, Vashon, WA, USA) were 25 mm thick × 50 mm wide × 50 mm long having an average surface roughness of 1.86 *μ*m (i.e., 0.007% of plate thickness); they were each used only once for MOB tests.

#### 2.3.4. Cylinder-on-Flat Indentation Tests

**Figure 2:**
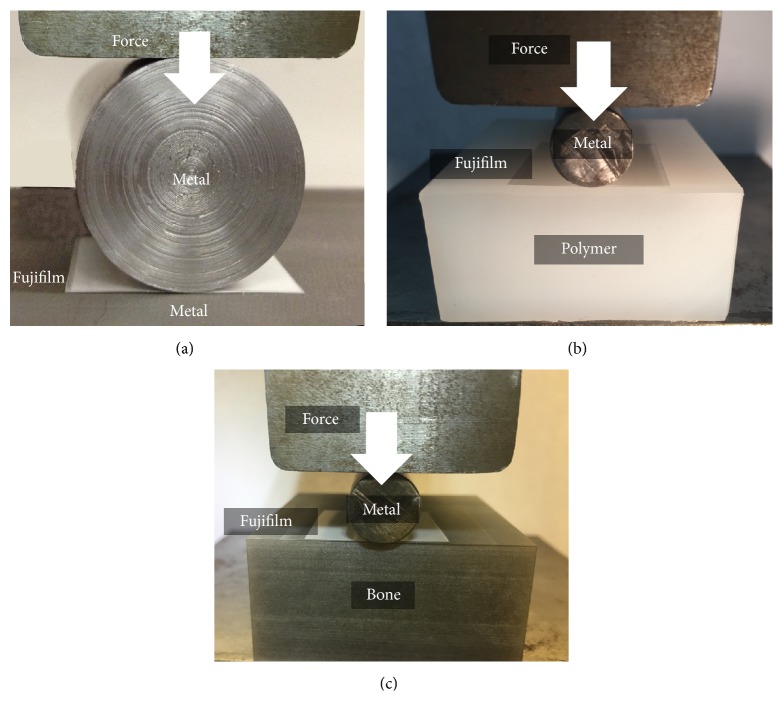
Experimental test setup. (a) Metal cylinder on a metal plate for MOM tests, (b) metal cylinder on a polymer plate for MOP tests, and (c) metal cylinder on a bone plate for MOB tests. A 0.2 mm thick sheet of Fujifilm is inserted at the interface.

A mechanical tester (Model #STM-50KN, United Testing Systems Canada Ltd, Concord, ON, Canada) with a 50 kN linear load capacity, 140 kN/mm frame stiffness, and +/− 0.1% accuracy had a built-in load cell and displacement transducer and was used for all tests (Figure 2). The investigation was conducted at an ambient temperature of 22°C. Metal cylinders were in turn placed on top of the Fujifilm, which rested on top of the metal, polymer, or bone plate sitting on the mechanical tester's base. Another steel block was mounted to the mechanical tester's load cell and was used to apply a vertical preload of 100 N to the top of the cylinder-on-flat to remove “mechanical slack.” Each metal cylinder was then loaded at 50 N/s in separate tests to 750 or 3000 N, the load was sustained for 60 s with only minimal material relaxation, and each cylinder was unloaded at 50 N/s. Each test was replicated 3 times to obtain an average. Forces corresponded to 1x and 4x body weight for a 75 kg person, as occurs at lower extremity joints during walking [23]. Force ramp-up was linearly elastic as shown by force-versus-displacement graphs, which had average coefficients of *R*^2^ = 0.97 (MOM), 0.98 (MOP), and 0.99 (MOB). Force rates, levels, and sustain times were chosen based on prior Fujifilm literature [1, 9, 11, 13, 15]. Note that because of the length of the flat substrates as described above, 75 mm of cylinder length was in contact with the flat substrates for MOM indentation tests, but 50 mm of cylinder length was in contact with the flat substrates for MOP and MOB indentation tests.

#### 2.3.5. Fujifilm Image Analysis

Fujifilm contact patches on the indicator layers from the indentation tests were scanned in bitmap format at 1200 dpi (dots per inch, or pixels per inch). Next, images were imported into Paint (Microsoft Corp, Redmond, WA, USA), the contact widths were measured in pixel units at 7 locations that were 3 mm apart lengthwise and then averaged, and the final results were converted to mm units.

### 2.4. Computational Analysis: Contact without and with Fujifilm

**Figure 3:**
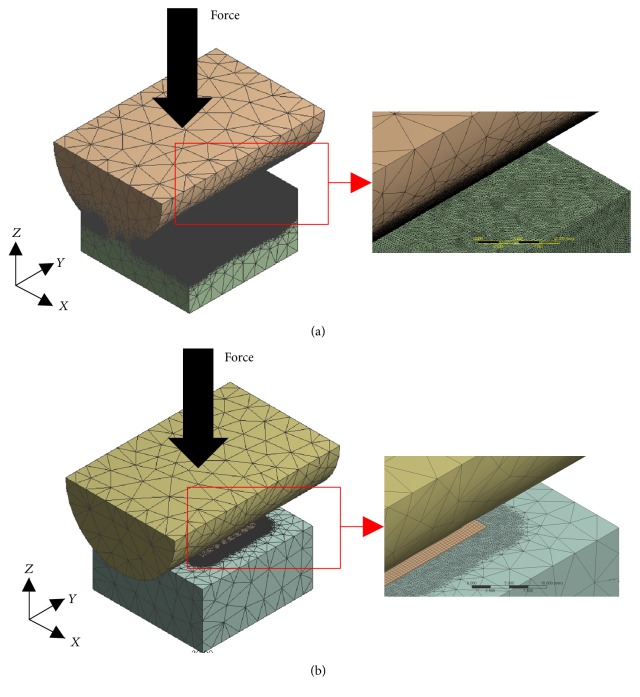
FE modelling and analysis configurations. (a) Contact without Fujifilm and (b) contact with Fujifilm. The same geometries, meshes, and boundary conditions were used for all material combinations of MOM, MOP, and MOB, whilst friction coefficients and material properties were varied accordingly. The area immediately around the contact zone appears dark because the mesh is very dense.

Finite element (FE) modelling and analysis were performed using Workbench 17.0 (ANSYS Inc., Canonsburg, PA, USA) to double-check the accuracy of the theoretical and experimental analyses (Figure 3). Specifically, 6 test cases were evaluated using one load level (i.e., 750 N), one cylinder diameter (i.e., 50.8 mm), all three material combinations (i.e., MOM, MOP, and MOB), and both Fujifilm conditions (i.e., without and with). The geometry of the FE models included a half-cylinder with a 50.8 mm diameter, Fujifilm with a 0.2 mm thickness, and a plate having 25 mm thickness × 50 mm width × 50 mm length, as done above. Note that a half-cylinder was used, rather than a full cylinder, in order to greatly reduce computational time and to ensure even load distribution. The meshing of the FE models comprised tetrahedral elements (i.e., Solid 187) for the half-cylinder and plate, as well as quadrilateral elements (i.e., Shell 181) for the Fujifilm; a denser mesh was used in the region immediately around the contact zone. The total number of elements and nodes, respectively, were 58792 and 99515 (half-cylinder), 89516 and 150368 (plate), and 3055 and 3164 (Fujifilm). The boundary conditions of the FE models involved assigning different friction coefficients *μ* for Fujifilm contact with all other surfaces (*μ* = 0) [15, 16], MOM contact (*μ* = 0.420) [26], MOP contact (*μ* = 0.045) [27, 28], and MOB contact (*μ* = 0.080) [29]. The materials properties of the FE models were set for elastic modulus *E* and Poisson's ratio *ν* for steel (*E* = 210 GPa, *ν* = 0.31), UHMWPE (*E* = 0.9 GPa, *ν* = 0.4), and artificial cortical bone (*E* = 16.7 GPa, *ν* = 0.3) as done above [3, 21, 22, 24, 25], as well as for Fujifilm (*E* = 0.1 GPa, *ν* = 0.45) [14–16]. The constraints of the FE models were attributed to the cylinder to allow for deformation (i.e., no constraints in *x*, *y*, or *z* directions), the plate bottom to mimic resting on a rigid foundation (i.e., constrained in the *x*, *y*, and *z* directions), and the Fujifilm to simulate no crinkling or lift-off (i.e., constrained in the *z* direction at the edges only). The 750-N load was applied directly to the top of the steel half-cylinder to create a compression depth at the cylinder-on-flat interface without and with Fujifilm. The maximum compression depth for Fujifilm cases was fixed at 0.2 mm (i.e., film thickness), since it was assumed that the much softer film would be fully compressed before the start of any compression of the much stiffer underlying plate material. Finally, contact width was defined as the total width for which there was physical touching of the cylinder with the plate and/or Fujifilm.

## 3. Results

### 3.1. Contact Patches

**Figure 4:**
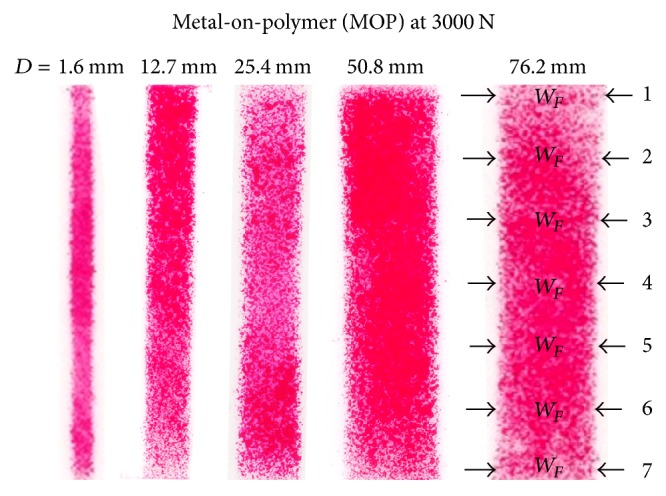
Fujifilm contact patches at 3000 N for MOP experiments. Similar results were obtained for MOM and MOB experiments. Contact width *W*_*F*_ was measured at locations 1 to 7 along the length of each patch.

**Figure 5:**
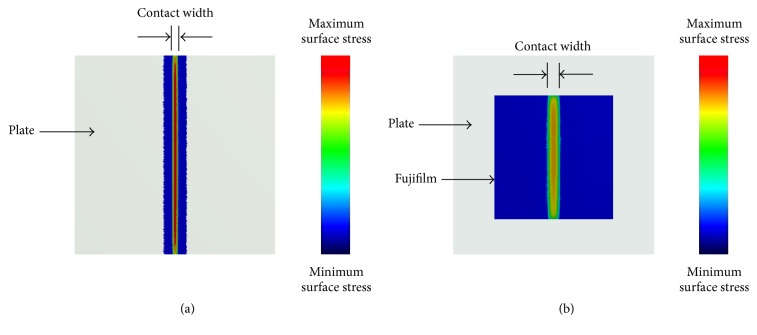
FE modelling and analysis contact patches. (a) Contact without Fujifilm and (b) contact with Fujifilm. These results are for one load level (750 N), one half-cylinder size (50.8 mm diameter), and one material combination (MOP). Similar results were obtained at the same load and for the same cylinder size for MOM and MOB simulations. Contact width was defined as the total width for which there was physical touching of the cylinder with the plate and/or Fujifilm, which ended just prior to the start of the minimum surface stress zone.

Magnified images of typical Fujifilm contact patches obtained during experiments are shown (Figure 4). Contact patches were relatively uniformly dense at each location along the length, but did not always have a perfectly uniform edge and showed some rough edges and noncontiguous patches. Similarly, contact patches predicted from FE modelling and analysis showed comparable features without and with Fujifilm (Figure 5).

### 3.2. Contact Widths

**Figure 6:**
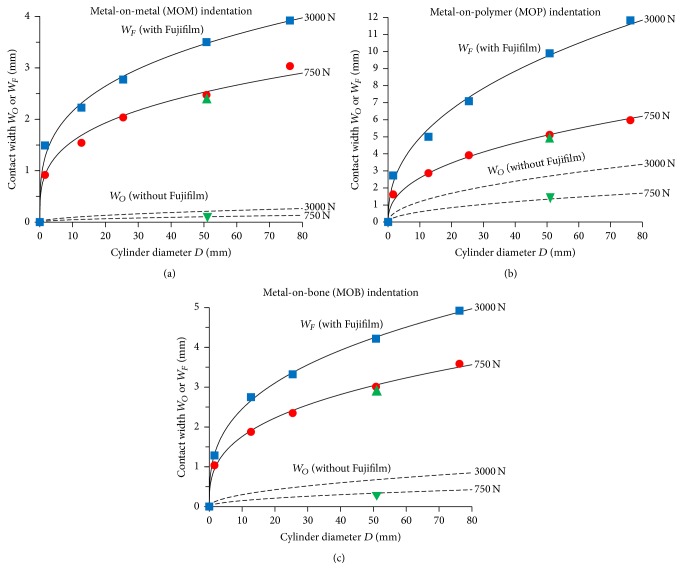
Contact width results. (a) MOM indentation, (b) MOP indentation, and (c) MOB indentation. *W*_*O*_ is true contact width determined by Hertzian theory without Fujifilm (dashed lines, - - -). *W*_*F*_ is measured contact width determined experimentally with Fujifilm, such that data (blue square, red circle) were fitted with power law lines-of-best-fit (solid lines, —) which yielded high coefficients of determination (*R*^2^ > 0.99). Standard deviations for experiments are not shown to avoid graph clutter, but they averaged 9.3% (MOM), 3.4% (MOP), and 7.9% (MOB) of the mean, indicating highly reproducible results. By definition, a cylinder with a 0 mm diameter would create contact widths *W*_*O*_ and *W*_*F*_ of 0 mm. FE analysis was done to double-check the accuracy of Hertzian theory and experiments at 750 N for a 50.8 mm diameter cylinder, yielding excellent agreement for test cases with (green triangle) and test cases without (green downward triangle) Fujifilm.

Contact width graphs without and with Fujifilm are provided over a range of cylinder diameters and compressive forces (Figure 6). Theoretical and experimental data both showed a gradual nonlinear rise in contact widths *W*_*O*_ and *W*_*F*_ as cylinder diameter *D* increased, whilst graphs shifted up or down depending on the compressive force. The Hertzian contact formula described above for *W*_*O*_ was reminiscent of the power law lines-of-best-fit running through experimental data, yielding the equations *W*_*F*_ = 0.1778 × *F*^0.2273^ × *D*^0.2936^ for MOM, *W*_*F*_ = 0.0449 × *F*^0.4664^ × *D*^0.4201^ for MOP, and *W*_*F*_ = 0.1647 × *F*^0.2397^ × *D*^0.3394^ for MOB, where *W*_*F*_ is contact width measured by Fujifilm, *F* is compressive force, and *D* is cylinder diameter. These lines-of-best-fit yielded high coefficients of determination (*R*^2^ > 0.99) for experimental data. Computational FE analysis for the 6 test cases agreed with the data from Hertzian theory (without Fujifilm) and experiments (with Fujifilm), respectively, to within 3.3% and 1.4% (Figure 6); thus, the appropriateness of these methodologies was validated.

### 3.3. Contact Width Error

**Figure 7:**
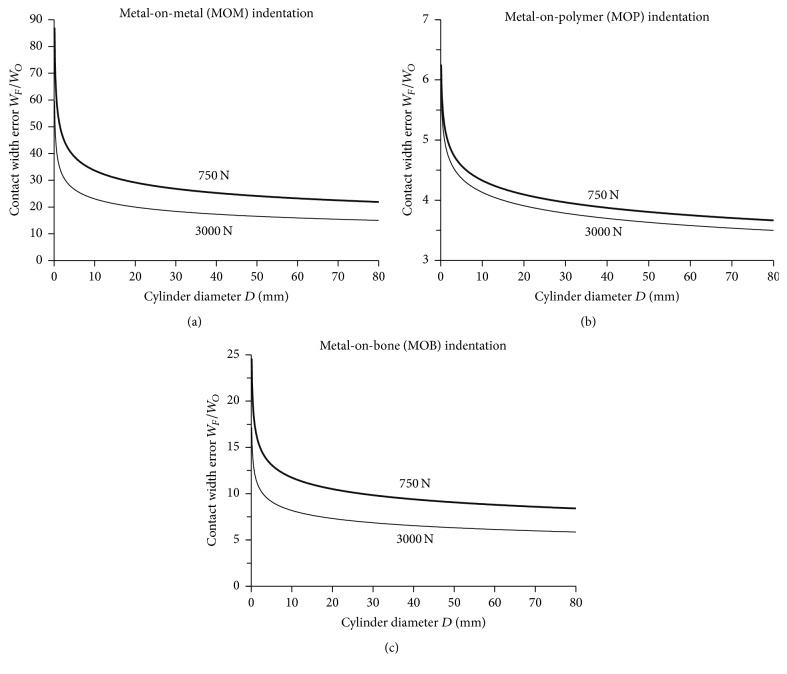
Contact width error. (a) MOM indentation, (b) MOP indentation, and (c) MOB indentation. *W*_*O*_ is true contact width determined by Hertzian theory without Fujifilm. *W*_*F*_ is contact width determined experimentally with Fujifilm.

Contact width error ratio *W*_*F*_/*W*_*O*_ was computed for the full range of compressive forces and cylinder diameters (Figure 7). Trends showed a nonlinear relationship of error ratio versus cylinder diameter, whilst the graph shifted down with higher compressive force. Error ratio was highest at small cylinder diameters and decreased asymptotically to a steady-state value at large cylinder diameters. Error ratio was highest for MOM, followed by MOB, and finally MOP because of the decreasing mechanical stiffness of metal, bone, and polymer flat substrates. The reason is that, for a given force and diameter, a lower mechanical stiffness of the flat substrate would permit the formation of a contact area that is larger in size relative to any Fujifilm measurement error which remains constant in size around the edges of the contact area.

## 4. Discussion

### 4.1. Comparison to Prior Studies

No previous investigations theoretically, experimentally, and computationally determined Fujifilm measurement error due to film thickness for two-body contact for several material combinations relevant to orthopaedics. However, Bachus et al. [13] showed that Fujifilm contact area measurement error for a 405 mm^2^ circular area under a 1250 to 4250 N load could range from a 1% overestimate to a 27% underestimate, depending on the image processing technique used. Matsuda et al. [30] evaluated the effect of placing Fujifilm between a flat-ended steel indenter with a circular area and a polymer plate supported by a steel plate. Their Fujifilm had a sensing range of 1 to 9.8 MPa and underestimated the contact area under low loads because the film could not detect areas where pressure was below 1 MPa. Fujifilm contact area underestimations for total knee replacements have been reported as 35% by Szivek et al. [7] and 11 to 36% by Harris et al. [11], which were also attributed to the lower pressure threshold limit of the film. Liau and coworkers [16] applied Hertzian theory, FE analysis, and experiments on a commercially available MOP total knee replacement over a range of loads and loading rates; they found that Fujifilm always overestimated the true contact area by 20–25% (for high congruency cases) and 14–77% (for low congruency cases) due to its thickness. Hoffman et al. [31] did theoretical, computational, and experimental analyses for a MOM ball-on-plate and found that Fujifilm overestimated true contact widths by a factor of 1.35–3 times. Similarly, the current study showed that the errors caused by Fujifilm thickness always substantially overestimated the true contact width, depending on the particular materials, geometries, and forces involved (Figure 7).

### 4.2. Clinical and Biomechanical Implications

Current findings show that Fujifilm measurement error due to thickness is extremely problematic for a noncongruent interface. It may also be potentially problematic for nearly congruent articulating bodies where perfect mating is never achieved because the gap between the bodies is less than Fujifilm thickness. This can occur in human knee joints whose congruity has been reduced due to a loss of meniscus [32, 33] and for total knee replacements designed to have small contact areas between the metal femoral and polymer tibial components at certain knee flexion angles [6]. Fujifilm-measured contact areas can then be grossly overestimated and the average and peak contact stresses will be greatly underestimated. Since contact stress is an important factor in the wear of human and artificial knee joints, accurate assessment of contact area is crucial [4, 6]. Consequently, when using Fujifilm for in vitro biomechanical testing, researchers should be aware of its measurement limitations.

Any area or pressure measurement technology in which a thin film is interposed between two articulating surfaces may show behavior similar to that described here for Fujifilm. K-scan, for example, is a widely used resistive ink sheet interposed at an articulating interface [10, 34]. It is commercially available, is suitable for static and dynamic real-time measurements of contact area and pressure, has a spatial resolution of about 27 data points/cm^2^, and has shown superior accuracy compared to Fujifilm in biomechanical applications [11–13, 30, 35, 36]. Nonetheless, K-scan's 0.1 mm thickness still predisposes it to the same type of measurement error shown here for Fujifilm. The degree to which K-scan artefact would follow the same trend demonstrated presently would still need to be conclusively shown in a future investigation.

Experimental measurement error due to the thickness of interposing layers may potentially be circumvented by methods which do not interfere with interfacial contact mechanics. Ultrasound has been used to study in vitro contact areas of total knee replacements, although ultrasound wavelength and beam thickness restrict image resolution [1, 9, 37–40]. The “interference method” calculates in vivo or in vitro contact area based on the relative positions of the mating bodies and their premeasured surface geometries, although surface deformation during articulation is not incorporated [41–43]. Radiography has been used in vivo to image barium-filled joints to estimate contact area, although the curvature of joint surfaces and lack of pressure measurement capabilities are limitations [44, 45]. These other procedures are still relatively expensive and complex to use and have their own limitations; thus, the low cost, availability, quick results, and easy use of Fujifilm still make it an appealing method for orthopaedic biomechanics researchers.

### 4.3. Case Study: Total Knee Replacement

**Figure 8:**
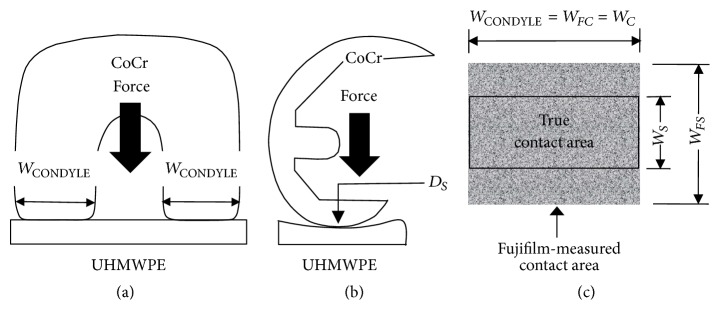
Case study of Fujifilm contact area measurement error predicted for one condyle of a total knee replacement. (a) Coronal plane geometry with the total knee replacement in 90° flexion, (b) sagittal plane geometry with the total knee replacement in 90° flexion, and (c) true contact area versus Fujifilm-measured contact area. *W*_*C*_, *W*_*S*_ are true contact widths in coronal and sagittal planes; *W*_*FC*_, *W*_*FS*_ are Fujifilm-measured contact widths in coronal and sagittal planes.

Fujifilm measurement error caused by film thickness can be estimated for a commercially available knee implant, such as the Omnifit Series 7000 TKR (Osteonics, Allendale, NJ, USA) [6]. First, assume that the cobalt-chrome (CoCr) femoral condyles in the coronal plane are almost flat with only slightly rounded edges (Figure 8(a)), but they have a curvature diameter of *D*_*S*_ in the sagittal plane at the tibiofemoral interface (Figure 8(b)). Thus, each condyle can be modelled as a CoCr cylinder with a fixed width *W*_CONDYLE_ that compresses a flat tibial layer made from UHMWPE. Second, for each condyle this will yield true contact widths of *W*_*C*_ and *W*_*S*_ in coronal and sagittal planes. It will be assumed that Fujifilm will overestimate contact width only in the sagittal plane (i.e., *W*_*FS*_ > *W*_*S*_) (Figure 8(c)), but not in the coronal plane (i.e., *W*_CONDYLE_ = *W*_*FC*_ = *W*_*C*_) (Figure 8(c)), because of the cylinder-like geometry of the CoCr condyles (Figures 8(a) and 8(b)). This yields a contact area measurement % error = (Fujifilm contact area − true contact area)/(true contact area) × 100 = (*W*_*FC*_ × *W*_*FS*_ − *W*_*C*_ × *W*_*S*_)/(*W*_*C*_ × *W*_*S*_) × 100. Third, prior Fujifilm tests on a single CoCr condyle of the Omnifit implant undergoing 335 N during 90° knee flexion generated line-type contact with *W*_*FC*_ = 13.61 mm and *W*_*FS*_ = 2.35 mm [6]. Fourth, recall that the Hertzian equation for cylinder-on-flat contact is [3, 21, 22]

(2) $$\begin{array}{c} W_{O}=\sqrt{\left(\frac{8FD_{cyl}}{\pi L_{cyl}}\right)\left(\frac{1-\nu_{cyl}^{2}}{E_{cyl}}+\frac{1-\nu_{flat}^{2}}{E_{flat}}\right)}, \end{array}$$

where *W*_*O*_ is true contact width in the sagittal plane without Fujifilm (i.e., *W*_*S*_), *F* is compressive force of 335 N, *D*_cyl_ is CoCr condyle curvature diameter of 36.5 mm in the sagittal plane during 90° knee flexion [1, 6], *ν*_cyl_ and *ν*_flat_ are CoCr and UHMWPE Poisson's ratios of 0.31 and 0.4 [22], *E*_cyl_ and *E*_flat_ are CoCr and UHMWPE elastic moduli of 210 and 0.9 GPa [24], and *L*_cyl_ is *W*_CONDYLE_ of 13.61 mm. This generates a *W*_*S*_ = 1.46 mm. Consequently, the contact area measurement % error = (*W*_*FC*_ × *W*_*FS*_ − *W*_*C*_ × *W*_*S*_)/(*W*_*C*_ × *W*_*S*_) × 100 = (13.61 × 2.35 − 13.61 × 1.46)/(13.61 × 1.46) × 100 = 61%. This value agrees with the study by Liau et al. [16], who found that Fujifilm consistently overestimated the true contact area for a MOP total knee replacement by 20–77% depending on the particular test conditions. Despite simplifications, this case study underlines the basic premise of this “proof of principle” study by illustrating Fujifilm measurement error due to film thickness.

### 4.4. Limitations

Firstly, this study investigated only two-dimensional line-type contact, although biomechanical investigators also use Fujifilm for complex three-dimensional interfaces of human and artificial joints [1, 5–11, 15, 32, 33, 37]. Secondly, although only two loads were evaluated, they did simulate clinical loads for the single-legged stance phase of walking for a 75 kg person (i.e., 1x and 4x body weight) [23, 46, 47]. Thirdly, theoretical contact widths *W*_*O*_ were orthogonal projections that ignored the slight curvature at the cylinder-on-flat interface caused by the cylinder, whereas experimental *W*_*F*_ measurements were made by “unfolding” the Fujifilm squares which had followed the cylinder curvature during the indentation tests [3]. This might have exaggerated current values for Fujifilm measurement error in the lower test range (i.e., *D* < 10 mm) when contact width was comparable to cylinder diameter, which possibly indicates some minor plastic deformation [22]. Nonetheless, as mentioned earlier, linear elasticity was maintained overall, based on average force-versus-displacement *R*^2^ values all being above 0.97. Finally, point-type, circle-type, and ellipse-type contact were not examined as found in some human and artificial joints [3], although at present the line-type contact examined simulated the articulations observed for various total knee replacements [1, 6] and fracture fixation devices as a “proof of principle.” Moreover, ball-in-socket articulations found in artificial hip prostheses were not explicitly investigated. Such articulations could potentially amplify the Fujifilm measurement artefact described presently because the highly conforming nature of the two surfaces would “squeeze” the Fujifilm even more readily in the narrow physical space around the actual contact zone. This could cause Fujifilm to erroneously register physical contact even more prematurely. Future investigators could extend the current analysis to more congruent articulations of some artificial and human joints [1, 6–12, 32, 33, 41, 43–45].

## 5. Conclusions

To the authors' knowledge, this is the first report to explore Fujifilm measurement error due to film thickness for a number of material combinations relevant to orthopaedics. Biomechanical measurement error due to the physical thickness of Fujifilm for a cylinder-on-flat articulation was quantified by comparing Hertzian contact theory with a series of Fujifilm experiments, both of which were confirmed for several test cases by FE modelling and analysis. The “proof of principle” results may be particularly important for nonconforming line-type articulations, as may occur for some types of total joint replacements, fracture fixation techniques, and other orthopaedics applications. However, a case study showed that measurement error is still present even for congruent interfacial articulations. Finally, the real issue this paper highlights is not about the relative size of the contact width with respect to the thickness of the Fujifilm, but rather the real concern has to do with what is happening at the periphery of the contact zone. This means that, in the presence of Fujifilm, if a physical space (i.e., gap) between two conforming articulating surfaces is less than the thickness of the Fujifilm, the Fujifilm will be physically “squeezed” inside that gap and then erroneously register that physical contact has occurred between those two surfaces. However, in the absence of Fujifilm, there is in fact no physical contact between the two articulating surfaces at the periphery of the actual contact zone.

## Data Access

The corresponding author can be reached to obtain original data.
